# Making the links: do we connect climate change with health? A qualitative case study from Canada

**DOI:** 10.1186/1471-2458-13-208

**Published:** 2013-03-08

**Authors:** Francesca S Cardwell, Susan J Elliott

**Affiliations:** 1 Department of Geography and Environmental Management, Faculty of Environment, University of Waterloo, 200 University Avenue West, Waterloo, ON, Canada; 2 School of Public Health and Health Systems, Faculty of Applied Health Sciences, University of Waterloo, 200 University Avenue West, Waterloo, ON, Canada

**Keywords:** Health, Climate change, Risk perceptions, Qualitative methods, Canada

## Abstract

**Background:**

Climate change has been described as the biggest global health threat of the 21^st^ century. Typically framed as an environmental issue, some suggest this view has contributed to public ambivalence and hence a lack of public engagement. The lack of understanding of climate change as a significant environmental health risk on the part of the lay public represents a significant barrier to behaviour change. We therefore need to think about reframing the impact of climate change from an environmental to a health issue. This paper builds on calls for increased understanding of the public’s views of human health risks associated with climate change, focusing on facilitators and barriers to behaviour change.

**Methods:**

Semi-structured in-depth interviews (n = 22) with residents of the Golden Horseshoe region of Southern Ontario were conducted between August 2010 and January 2011. Topics included individual and community health, climate change, and facilitators and barriers to behaviour change.

**Results:**

Few participants recognized the role of the environment in the context of either individual and community health. When asked about health concerns specific to their community, however, environmental issues were mentioned frequently. Health effects as possible impacts of global environmental change were mentioned by 77% of participants when prompted, but this link was not described in great detail or within the context of impacting their communities or themselves. Participants were willing to act in environmentally friendly ways, and possible incentives to undertake behaviour change such as decreasing cost were described. Health co-benefits were not identified as incentives to engaging in mitigative or adaptive behaviours.

**Conclusions:**

The results support recent calls for reframing the impact of climate change from an environmental to a public health issue in order to increase public engagement in adaptive and mitigative behaviour change. While previous research has touched on public awareness of the human health risks of climate change, we have further explored the attitude-action link through the examination of facilitators and barriers to behaviour change.

## Background

Most of us are now in agreement that environmental sustainability is increasingly threatened by changes to the natural environment that will significantly affect human and ecosystem health [1,2]. Climate change has been described as one of the most threatening of these changes [3]. Further, the Intergovernmental Panel on Climate Change (IPCC) (2007) tells us that human activities (such as transportation, industrial activity, land-use change, and daily consumer choices) are the primary factors affecting changes to the earth’s climate (e.g., altered surface temperatures, precipitation changes, sea-level rise, and changes in the frequency and magnitude of extreme weather events [4]). Relatedly, climate change has been dubbed the biggest global *health* threat of the 21^st^ century [3], resulting in, for example, changes in the range and transmission of infectious disease, food insecurity, health impacts of air pollution and temperature stress, reduced access to safe water, and related psychosocial impacts [2,3].

While climate change has been, for many years, framed as an important environmental issue, this view has resulted in overall public ambivalence and contributes to a lack of public engagement [5]. The way the public frames an issue (how it is mentally organized and communicated with others) can influence public understanding and encourage (or discourage) behaviour change related to the problem [5]. Linking a complex topic (in this case, the impacts of climate change) to what is already valued as important (for example, health risks such as asthma, infectious diseases) may be a useful mechanism to bring the issue closer to home thereby increasing its relevance to the public, and potentially encouraging behaviour change [6,7].

There has been recent increased recognition of climate change as a health issue by both the lay public and public health officials [1,5,6]. For example, a 2001 survey of the public in 30 countries showed that almost a third of respondents named human health as their greatest concern when provided with a list of potential climate change impacts [8]. In a recent large-scale quantitative study using nationally representative surveys in the US, Canada, and Malta between 2008 and 2009, Akerlof et al. found that the majority of people in all three countries believe climate change poses significant risks. Further, one half of Canadians said people are already being harmed. While Canadian respondents identified that climate change poses health risks, relatively few people could answer open-ended questions linking climate change to health; only 10% of Canadians named climate change without prompting when asked what environmental problem or hazard poses the greatest risk to the nation’s health [6]. Similar results in a small quantitative Canadian study focused on residents of the province of Alberta [9].

In Akerlof et al.’s large-scale quantitative study, Americans were more likely to consider people in developing countries to be at risk from climate change, while Canadians identified themselves, their families and their communities as being vulnerable to at least moderate harm from climate change [6]. Other polls also indicate a difference in the way Canadians and Americans perceive climate change as a public health threat [10-12], indicating that there may be significant differences in how people in different countries perceive these issues [6].

Despite increased attention, the lay public does not yet fully comprehend the health implications of climate change. For example, about half of American survey participants responded “I don’t know” when asked the estimated number of current and future injuries, illnesses, and deaths due to climate change [13]. A lack of understanding or recognition of climate change as a significant risk represents an important barrier to behaviour change. For example, it has been demonstrated that Americans who view climate change as being harmful to people or who understand that it is a current threat (versus in the future and/or in distant places) are more likely to support climate policy and personal behaviour change [13,14].

Given the above, we suggest the need to reframe the impact of climate change from an environmental to a health issue, in order to encourage necessary behaviour change. Giving climate change a public health focus suggests that there is a need for behaviour change in order to both mitigate (ie. reduce greenhouse gas emissions) and adapt to the problem (ie. protect communities and people from current and future health related impacts) [5].

While these studies point us in the direction of a link between climate change, public health and behaviour change, the literature in this area is limited [6]. Indeed, Akerlof et al. suggest that “there has been relatively little research on public awareness and understanding of the human health impacts and risks associated with climate change, and almost none of the research has been published or synthesized in the academic literature” (p. 2560). Further, little attention has been focused on the attitude-action link; that is the link between perception of the risk and (mitigative) behaviour. Akerlof et al. (2010) go on to suggest we need more qualitative work in order to fully understand possible ways to reduce risks to individuals and communities [6]. We take this beyond understanding (perceived) risk to focusing on facilitators and barriers to (mitigative) behaviour change.

In this context, in order to answer the question “What are the knowledge, attitudes, and practices related to global environmental change and health of residents of the greater Hamilton area of the Canadian Golden Horseshoe region?”, we address the following research objectives:

1. to understand knowledge and attitudes of residents of the Canadian Golden Horseshoe region regarding global environmental change, with a particular focus on perceived health impacts of climate change;

2. to document actions taken by Golden Horseshoe residents to mitigate the health risks of climate change; and

3. to investigate potential behaviour change mechanisms.

## Methods

**Figure 1:**
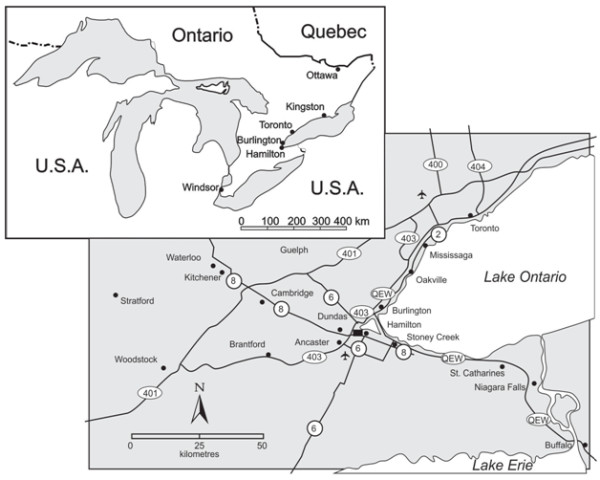
Study location - Southern Ontario, and the Golden Horseshoe Region.

This research took place in The Golden Horseshoe region of Southern Ontario, Canada, which is located on the western edge of Lake Ontario and stretches from Oshawa, east of Toronto, to Niagara Falls (Figure 1). One quarter of the Canadian population resides in this region making it both the most populous and heavily urbanized area in Canada [15]. Central in the Golden Horseshoe is Hamilton, Ontario. Historically, Hamilton has been known for its role in the steel manufacturing industry [16,17]. This has given the western end of the Golden Horseshoe a reputation of poor air and water quality, both locally and nationally [18,19]. In addition to industrial pollution, Hamilton receives cross-border air pollutants from American industry [18]. Consequently, community environmental concern in the region has been present since wartime industrial growth [20]. Recently, local concern and action has enhanced Hamilton’s air and water quality to bring it to levels similar to other Southern Ontario cities, although the stigma remains [19].

Participants were recruited from the greater Hamilton area of the Golden Horseshoe region, including neighbouring cities of Burlington and Brantford, and the town of Oakville. Hamilton, Ontario, consists of various residential communities including Ancaster, Dundas, Flamborough, Glanbrook, Hamilton, and Stoney Creek, and participants were recruited from various regions of the City including Dundas, and Stoney Creek. The greater Hamilton area of the Golden Horseshoe region was chosen as the research setting because of the area’s reputation for poor environmental quality, and the evidence of community concern and action centered around air and water quality [19,20]. In addition, communities in this region are sufficiently diverse in their socio-demographic variables to employ a purposive maximum variation sampling method (see Additional file 1).

The McMaster University Research Ethics Board granted ethical approval prior to the start of the research. Research participants (n = 22) were recruited using a maximum variation sampling method (see Additional file 2 for Participant Demographic Breakdown). Community notification boards (in coffee shops, libraries, and community centres) and local free classified advertising websites (craigslist.ca and kijiji.ca) were used to recruit participants. All respondents to the research advertisement that consented to participation were included in the research (n = 15 from online sources, n = 1 from a poster on a community notification board). Further, snowball sampling was used to complete the sample and to attempt to fill any gaps in participant demographics. Recruitment continued throughout interviewing, until saturation of themes was reached. Participants were told they would be asked about health and environmental issues in their community, and were offered a $10 grocery gift card for participating. Semi-structured in-depth interviews ranged from 30 to 75 minutes and were conducted between August 2010 and January 2011. Participants were assured they would remain anonymous, and that their personal details would remain confidential. The interview schedule covered a range of topics including individual and community health, health behaviours, global environmental change, climate change and global warming, environmental behaviours, and facilitators and barriers to behaviour change (see Additional file 3). Interviews were digitally recorded with participant permission, and transcribed verbatim for subsequent exploratory thematic analysis using NVivo9. At the end of the interviews, member checking was employed and inter-rater reliability of the transcript coding was assessed to ensure rigour, with a documented agreement of 95% between the primary researcher and a second coder.

## Results

Twenty-two residents of the greater Hamilton area of The Golden Horseshoe region participated in the research. The participants ranged in age (at the time of research) from 21 to 68, and the sample included both males (n = 6) and females (n = 16). Participants exhibited a range of sociodemographic variables, including employment status, education, political views, level of community involvement, and country of birth (see Additional file 2 for Participant Demographic Breakdown).

Four primary themes emerged from the in-depth interviews: views of individual and community health; knowledge around climate change; attitudes toward climate change; and individual behaviours and facilitators and barriers to behaviour change. The results are reported using participant quotes and tables. The tables present the themes described in response to interview questions, the number of participants that identified each theme, and the number of times it was mentioned throughout the interviews.

### Views of individual and community health

When asked ‘what does health mean to you?’ respondents most often reported some combination of physical (e.g. exercise) and mental health (e.g. stress) (Table 1). Aspects of physical health (food, diet and exercise) were identified most frequently, and often in combination:

**Table 1:** Perceptions of what makes a person healthy

**Definition of Health**	**Number of participants (% of the total)**	**Mentions (% of the total)**
Physical	22 (100)	49 (45)
Exercise	14 (64)	19 (18)
Food and Diet	12 (54)	21 (19)
Disease/Illness/Sickness	6 (27)	6 (6)
Genetics	2 (9)	3 (3)
Mental Health	13 (59)	23 (21)
Stress	5 (23)	7 (6)
Social Factors	6 (27)	8 (7)
Environment	5 (23)	10 (9)
Spirituality/Religion	2 (9)	2 (2)
Other	7 (32)	9 (8)
*Total*	* 22	108 (100)

* This is not equal to the sum of the numbers in the column due to multiple responses.

"Well I figure there are several things. Diet, exercise, and your mental health. (Interview 18, Male)"

Social aspects of health (e.g. social isolation) were also reported by more than a quarter of the sample:

"We don’t even have to pick up the phone to dial people, we just text … and really you know that is kind of sad. You are even losing the personal, psychological impact from interacting with people, making you feel more awkward when you do make new friends. You feel more socially isolated. (Interview 17, Female)"

Only 23% (n = 5) of participants reported the ‘environment’ as part of their definition of health.

Alternatively, when asked what makes a *community* healthy, 32% (n = 7) of participants identified environmental factors. Pollution, specifically air quality, was most frequently identified as an environmental determinant of community health:

"… air quality is important in a community to be healthy, and there are some areas in Hamilton that have lousy air quality. (Interview 16, Female)"

When participants were asked about specific health concerns in their own communities (Table 2), environmental issues were identified by 73% of participants (n = 16). Concern was limited to pollution (notably water and air). Few specific health concerns were described, however asthma and respiratory problems were mentioned as related to air pollution:

**Table 2:** Top five community health concerns

**Health Concern**	**Number of Participants (% of total)**	**Mentions (% of the total)**
Environment - Total	16 (73)	39 (23)
Air Pollution	12 (54)	26 (15)
Garbage	5 (23)	7 (4)
Drinking Water	4 (18)	6 (3)
Accessibility to Services (ie. medical, education, housing)	11 (50)	24 (14)
Transportation	9 (41)	31 (18)
Obesity/Nutrition/Physical Activity	9 (41)	25 (14)
Poverty	6 (27)	15 (9)
*Total*	* 22	173 (100)

* This is not equal to the sum of the numbers in the column due to multiple responses.

"In my community, I would say industrial pollution is probably another big issue too because I think from having grandchildren, I notice that a lot of younger kids now, I would say a high percentage either have asthma or they are on puffers. (Interview 13, Female)"

### Knowledge around climate change

When asked ‘what does the term *global environmental change* mean to you?’ respondents spoke of various causes and impacts of global environmental change, climate change and global warming (Table 3). Health effects were reported by the majority (77%) of respondents, however describing the perceived health effects in extended detail was less common. When detail was given, health risks related to air pollution (n = 8), sunburn/cancer (n = 5), heat (n = 5), and natural disasters (n = 5) were most frequently identified (Table 4).

**Table 3:** Knowledge around climate change

**Theme**	**Number of participants (% of the total)**	**Mentions (% of the total)**
Health effects	17 (77)	49 (21)
Weather/temperature change	15 (68)	22 (9)
Glaciers/ice caps	14 (64)	22 (9)
Pollution (general)	13 (59)	31(13)
Natural disasters	12 (54)	23 (10)
Ozone hole	11 (50)	16 (7)
Greenhouse gas emissions	8 (36)	16 (7)
Wildlife	8 (36)	14 (6)
Natural cycles	7 (32)	18 (8)
Deforestation	5 (23)	12 (5)
Sea-level rise	4 (18)	4 (2)
Rainforests	2 (9)	4 (2)
Storms	2 (9)	3 (1)
Water	2 (9)	3 (1)
Total	* 22	237 (100)

* This is not equal to the sum of the numbers in the column due to overlap in responses.

**Table 4:** Perceived health impacts

**Health Impact**	**Number of participants (% of the total)**	**Mentions (% of the total)**
Air pollution	8 (36)	11 (20)
Sunburn/cancer/UV exposure	5 (23)	7 (13)
Heat	5 (23)	6 (11)
Natural disasters	5 (23)	5 (9)
Food security/safety	2 (9)	2 (3)
Spread of infectious diseases	1 (5)	1 (2)
Other	10 (45)	22 (41)
Total	* 22	54 (100)

* This is not equal to the sum of the numbers in the column due to overlap in responses.

"I think with the hotter summers and everything, more people are being affected, like infants could get heat stroke, and the elderly could be affected. As an average person, I don’t think it really has hurt us that much. (Interview 10, Male)"

Fifteen (68%) participants identified weather changes as an expected impact of global environmental change. More specifically, the possibility of both temperature increases and decreases were described:

"I think our winters are the same as they were, in fact sometimes even colder, and if it was global warming, wouldn’t they be warmer? Our summers have been cool lately. So I don’t know where this global warming is happening, but I don’t feel it. (Interview 20, Female)"

Melting of ice, ice caps, icebergs, glaciers, the North Pole, and the Arctic was reported by sixty four percent (n = 14) of participants: *Up north like the North Pole, it is melting. (Interview 21, Female)*

In addition, the general presence of pollution (air, water, or litter) was described by 13 participants (59%): *Well the water pollution is pretty bad too, you know. (Interview 13, Female)*

Finally, natural disasters, such as tsunamis, earthquakes, floods, storms, and volcanoes were identified in 12 interviews (54%). Some respondents described the floods occurring at the time of interviews (late 2010/early 2011) in Pakistan, Australia, Brazil and the Philippines:

"When you think of three floods in one week… for example, the Brazil one, they thought three hundred died, well it is up to five hundred now, so the flooding is becoming larger. (Interview 22, Male)"

There was also significant confusion around the meanings of the terms global warming, global environmental change and climate change in discussions with respondents:

"I think global warming is everything being affected, where climate change is more or less just your temperatures. (Interview 10, Male)"

Another respondent, when asked about global warming, immediately responded with the term climate change, indicating the perceived interchangeability of the terms:

"R: So you mentioned global warming… what does that mean to you?"

"W: Climate change. (Interview 4, Female)"

When asked if they could differentiate between climate change, global warming and environmental change, 41% of participants (n = 9) stated that they are interchangeable. Although 32% (n = 7) of respondents identified a difference between the terms, they could often not articulate it:

"R: You used the term “global warming”. Do you see a difference between those terms?"

"W: Global warming, climate change. Well I know there is one. (Interview 1, Female)"

Finally, participants were asked if they could identify environmental resources, organizations, and programs in their communities. Awareness was limited, as only 41% (n = 9) of participants could identify at least one program or resource. The programs were not described in detail, or were only identified by name. Of the participants that identified resources, three said that they participate, while others either reported they did not participate (n = 4), or did not state either way. Eight participants said they had heard of resources but could not identify their names or what they do, while 5 stated they did not know of any.

### Attitudes toward climate change

Throughout the interviews a number of dominant attitudes emerged (Table 5). Many participant responses (82%) demonstrated varying degrees of concern toward global environmental change, climate change and global warming. Although some participants described personal worry, others stated they are not concerned:

**Table 5:** Attitudes toward global environmental change

**Attitude**	**Number of participants (% of the total)**	**Mentions (% of the total)**
Concern/worry	18 (82)	55 (22)
Cynical/skeptical	15 (68)	37 (15)
Humans responsible	14 (64)	23 (9)
Spatially distant	13 (59)	22 (9)
Too much hype/buzz word	8 (36)	16 (7)
Future time frame identified	8 (36)	10 (4)
Political agenda	6 (27)	25 (10)
Not my responsibility	6 (27)	16 (7)
Helplessness	5 (23)	10 (4)
There are benefits	4 (18)	7 (3)
Inaccurate representation in media	4 (18)	7 (3)
Just don't know	4 (18)	5 (2)
Don't want to think about it	2 (9)	4 (2)
Sad	2 (9)	3 (1)
Curious about the truth	2 (9)	2 (.9)
Optimistic things will get better	2 (9)	2 (.9)
Pessimistic about the future	2 (9)	2 (.9)
Total	* 22	246 (100)

* This is not equal to the sum of the numbers in the column due to multiple responses.

"R: [Do you worry] about climate change or global warming?"

"W: No I don’t, because I think in a few years, like I say, I think it will right itself. I am not sure if we keep polluting the air if it might take longer, but I am hoping that it can right itself. (Interview 16, Female)"

68% (n = 15) of participants described skepticism:

"Global environmental change. Well I am not sure. You hear global warming, which I don’t believe in. I think it is a load of horse manure actually. (Interview 18, Male)"

Despite feeling skeptical, many participants reported concern about related environmental issues. For example, one participant stated they were not worried specifically about climate change, but reported concern around air pollution and health:

"R: Are you concerned about your health and the health of your family related to these environmental issues?"

"W: Yes."

"R: What about climate change?"

"W: To the fact that the air is polluted… yea, I worry about that a lot. (Interview 16, Female)"

Thirteen participants’ (59%) responses suggest a distancing of personal responsibility and vulnerability around the causes and impacts of climate change. Consistent with distancing personal vulnerability, eight participants (36%) believed that the effects of climate change would be temporally distant and unlikely to impact their own lives. Participants refer to their children, grandchildren or the future being affected:

"So my expectations as to how the world is going to be two degrees warmer, 400 years from now, I can’t get involved. It is out of my interest level. (Interview 3, Male)"

In this vein, participants also reported feeling that the effects of environmental change were not their fault or responsibility. 27% (n = 6) of respondents identified other people or organizations accountable for environmental degradation:

"My son always says your generation ruined the environment. No it wasn’t my generation… I said, big businesses, corporations, ruined the environment, and then people were just not aware. (Interview 13, Female)"

### Behaviours related to the environment

Participants were asked what people could do to act in environmentally friendly ways. A variety of both individual and community level behaviours were identified (Table 6). By far, the most popular behaviours were recycling, reducing and reusing (described by 100% of participants):

**Table 6:** Environmental behaviours

**Behaviour**	**Number of participants (% of the total)**	**Mentions (% of the total)**
Recycle, Reduce, Reuse	22 (100)	228 (45)
Energy consumption	17 (77)	57 (11)
Water bottles/mugs/containers	10 (45)	20 (4)
Water	8 (36)	14 (3)
Cloth bags	5 (23)	15 (3)
Transportation-related	21 (95)	125 (24)
Green bin	15 (68)	59 (12)
Food-related	9 (41)	28 (5)
Public initiatives	8 (36)	11 (2)
Other	14 (64)	61 (12)
Total	* 22	512 (100)

* This is not equal to the sum of the numbers in the column due to multiple responses.

"But I do my part, right. I do the recycling. I recycle everything. People get mad at me, if they come to my house, because I will go through the garbage. (Interview 21, Female)"

Some participants described the inconvenience of recycling:

"I recycle all the time, but I don’t recycle all the time sometimes, because you… forget, or get a little bit lazy, just like everything else, but you try. (Interview 17, Female)"

Conserving energy by switching off or unplugging appliances, limiting the use of heating or air conditioning, reusing water bottles and reducing water consumption were other ways to recycle, reduce and reuse described by participants.

Transportation-related behaviours were reported by 95% (n = 21) of participants. Reducing car use was most frequently described, as participants identified driving less and using alternative methods of transportation. For example, walking or biking, limiting idling, using public transportation, and planning efficient car trips were discussed:

"If we are going to go to two or three places, we try to plan our routes so that we don’t use the car any more than we have to. (Interview 16, Female)"

Using the green bin (a local food and other biodegradable waste disposal system) was reported by 68% (n = 15) of participants. Although it was identified as beneficial, some participants said they did not participate in this initiative:

"To be honest, we don’t use the green bin. I don’t really even know what it is for. It is terrible… I think it is for like organic stuff? That just gets so disgusting, and I can’t handle maggots. Neither can my mom. So we just can’t do it. (Interview 1, Female)"

Participants were also asked what they believed would encourage environmentally friendly behaviours (Table 7). Four incentives were reported; reducing cost, increasing convenience, reducing time-consumption, and increasing enjoyment. Positive health co-benefits were not described by participants. Financial incentives were discussed by 19 respondents (86%). For example, using inexpensive public transit and turning the lights off to reduce electricity bills were described:

**Table 7:** Facilitators

**Facilitator**	**Number of participants (% of the total)**	**Mentions (% of the total)**
Cost	19 (86)	56 (68)
Convenience	10 (45)	15 (18)
Time	6 (27)	7 (08)
Enjoyment	3 (14)	4 (05)
**Total**	* 22	82 (100)

* This is not equal to the sum of the numbers in the column due to overlap in responses.

"I think everybody likes to save money, and I think you know it is important to turn off your computer, or turn off your lights, turn off any power sources that you may have that are just wasting energy for no reason, and I do that quite diligently in my house. (Interview 14, Female)"

Participants were also asked about barriers to behaviour change (Table 8). Convenience was described as a significant barrier by 13 participants (59%):

**Table 8:** Barriers to behaviour change

**Barrier**	**Number of participants (% of the total)**	**Mentions (% of the total)**
Convenience	13 (59)	40 (27)
Personal cost	12 (54)	22 (15)
Don’t know what to do	11 (50)	24 (16)
Lack of time	9 (41)	18 (12)
Negative economic impacts	8 (36)	13 (9)
Discomfort	7 (32)	8 (5)
Physical barrier	5 (23)	6 (4)
Already doing everything I can	4 (18)	5 (3)
I won’t make a difference	4 (18)	5 (3)
Habit, culture	4 (18)	4 (3)
Availability of options	3 (14)	5 (3)
**Total**	* 22	150 (100)

* This is not equal to the sum of the numbers in the column due to overlap in responses.

"If I could find more of the easy things to change I would. But not like drastic, turn my life upside down type of thing. (Interview 6, Female)"

In addition, not knowing how to act in environmentally friendly ways was identified. 50% (n = 11) of participants spoke of feeling overwhelmed and not knowing what to do:

"So for the environment, you know, somebody might tell you that it is better to do this, but then somebody will come up with something that says no it is not. Just nobody really knows, so just like information I think is a barrier. (Interview 10, Male)"

Participants (n = 7) also described discomfort as a barrier to pursuing environmental activities. Respondents did not want to sacrifice comfortable activities if they were not environmentally friendly, even with the knowledge of possible environmental impacts:

"Well I do take a cruise every year, and… it is a big gas guzzler - the ships - and I think in the winter when it is cold, I want to get away, and there is nothing else to do, and the plane, it pollutes the air, and I know that, but you do want to get away from the cold, and so you bite the bullet and you go and I feel bad about it. What else is there to do? (Interview 20, Female)"

## Discussion

This research uncovered a range of perceptions related to how global environmental change, particularly climate change, and health are understood by residents from the greater Hamilton area of the Golden Horseshoe region. The results reported here point to significant policy implications.

When asked specifically about individual health, few participants (n = 5) recognized the role of the environment without probing (Table 1). Similarly, when asked what factors influence a community’s health, the relationship between the environment and health was not often described. When identified, links were most often related to air quality and respiratory illness. Even in a region with a reputation for poor environmental quality and existing community environmental concern [19,20], the environment was not considered an important determinant of individual or community health.

This differed, however, when questioned about health concerns specific to their own community, as environmental issues were mentioned *most* frequently (by 16 participants [Table 2) compared with other health risks. This indicates that although participants are aware of environmental issues, they are not perceived as significant determinants of health unless they are considered immediately (or visibly) concerning (such as air pollution in Hamilton). Health risks from climate change were not identified in this discussion. This indicates that consistent with other research with Canadians [6], respondents do not yet consider climate change an important immediate health risk when unprompted.

When asked about the term ‘global environmental change’, 77% of participants (n = 17) mentioned health effects as possible impacts, particularly with respect to air pollution, sunburn and cancer, heat stress, and natural disasters. Clearly, then, the public is aware of the general link between global environmental change and health, but did not describe these links in great detail or within the context of specific impacts to their communities or themselves. When participants described health effects in more detail, air pollution was most commonly identified, perhaps because the study was conducted in an area where air pollution is already a significant health concern [19].

Emerging from this research is an indication of the lack of basic knowledge of global environmental change, climate change and global warming among the general public. While aware of basic terminology, detailed understanding is limited as demonstrated by confusion between the terms, and a self-stated lack of understanding. In the climate change literature and amongst the lay public, these terms have different meanings. Global environmental changes are large scale, potentially irreversible changes from human pressures (such as population growth) on the natural environment. Examples include climate change, as well as freshwater shortages, exhaustion of fisheries, and biodiversity loss [1]. According to the IPCC, climate change specifically refers to “a change in the state of the climate that can be identified by changes in the mean and/or variability of its properties, and that persists for an extended period, typically decades or longer” [4]. Although the terms climate change and global warming are often used interchangeably, climate change is most often used by the scientific community, while global warming is often used by the lay public, to primarily describe temperature increases associated with an enhanced greenhouse effect [8].

Confusion between terms and their meanings is therefore not surprising, as even credible sources (like academic studies) use these terms interchangeably. For example, Akerlof et al.’s (2010) study of public perceptions of global warming/climate change and human health uses different terminology across geographical regions. The Canadian and Maltese surveys used the term “climate change”, while the American survey used “global warming” [6].

The public’s lack of knowledge and confusion can also translate into misinformed attitudes. For example, concern was the most identified *possible* attitude. Some participants, however, described not feeling worried about climate change, but identified concern for other closely related environmental issues such as air pollution. A lack of concern could represent a significant barrier to voluntary behaviour change, reinforcing a need for basic information related to the contributing factors and risks of climate change in this region. At the same time, concern for other environmental health impacts amongst respondents shows promise for framing the impacts of climate change as public health risks. With this in mind, clear public health messages from trustworthy sources (e.g. public health agencies) are needed for the public to better understand and accept the clear links between climate change and health.

Consistent with other research [11,21], many respondents perceive the risks and impacts of climate change as temporally and spatially distant, detaching their own vulnerability and responsibility to take action. With this in mind, a public health frame would be useful to link climate change risks to local health impacts in order for climate change to be perceived as spatially and temporally relevant and context-specific. For example, in the Golden Horseshoe region of Southern Ontario, communication of risks should emphasize the local health impacts of heat stress, particularly on vulnerable populations such as the elderly and children [4,22]. This approach would be useful in encouraging public behaviour change (such as increasing use of public transit in order to reduce vehicular emissions), since research shows that people who understand climate change as a current and local threat are more likely to support policy and engage in personal actions [13,14].

Although a number of environmental behaviours were identified as possible actions (every respondent described some form of recycling, reducing and reusing), support decreased when behaviours were perceived as inconvenient, costly or time-consuming. When discussing reasons to participate in environmental behaviours (facilitators to behaviour change), participants described activities that are inexpensive (or could lead to financial benefits), convenient, take little time, or are enjoyable as more appealing. Interestingly, participants did not describe health co-benefits as incentives to engage in mitigative or adaptive behaviours. Reframing the impacts of climate change as health issues has the potential to increase awareness of another dimension of co-benefits, including cleaner air, healthier food, and pedestrian- and bicycle-friendly communities, providing more incentive to change behaviour or support climate policy. Recent research supports this recommendation; when climate change was introduced as a health problem with mitigation-related policy options that can lead to health benefits, a broad section of Americans responded positively [5].

When discussing barriers to behaviour change, half of these respondents described not knowing what to do. While this indicates a willingness to act in some participants consistent with previous research [11,21,23], a lack of knowledge on how to do so effectively represents a significant obstacle. Results also point to the need for inexpensive and comfortable options, in order to encourage individual behaviour change. Specifically, infrastructure that is affordable, efficient, and accepted by the public is needed to increase behaviour change where possible.

Finally, knowledge of and participation in environmental and/or health community resources, organizations and programs was limited in this sample. The lack of knowledge, combined with the attitude that individual behaviours do not make a difference, could indicate a lack of social capital and community connectedness amongst the residents of the greater Hamilton area of Southern Ontario. This is consistent with other research conducted in Hamilton, Ontario [19]. Increasing social capital (involvement in community networks) can encourage behaviour change and help overcome the sense of powerlessness associated with addressing environmental concerns.

While the purpose of this research was to qualitatively explore the public’s perceptions of climate change and health, the research is not generalizable on a wider scale and limitations exist. The sampling methodology could have restricted segments of the population from participation, and therefore certain opinions and perceptions could have been omitted. For example, the majority of participants (n = 15) were recruited using public classified advertisement websites (craigslist.ca and kijiji.ca), which could limit the sample frame to those with computer/Internet access, and those aware of online advertising websites. This limitation can be seen in the low proportion of men (n = 6) in the sample (a group that is often under-represented in social research), and the high proportion of participants with some level of post-secondary education (see Additional file 2 for full Participant Demographic Breakdown). Participants with unrepresented views from various cultural backgrounds or with lower sociodemographic status may therefore have been excluded. Consequently, some opinions may not be represented in the results. Although this was addressed through multiple recruitment methods (snowball sampling and posting the advertisement on multiple community boards across the study region), some populations could have been overlooked.

Further, participants from the Golden Horseshoe region represent only a small segment of the diverse Canadian population. The impacts of climate change will be perceived and experienced in very different ways in Southern Ontario compared with residents of other Canadian regions. For example, Ford & Smit identify how climate change in Canadian Polar Regions is expected to be amongst the greatest anywhere in the world. Climate change could severely disrupt human infrastructure (such as transportation routes and housing), and significantly impact livelihoods that rely on traditional hunting activities and traditional knowledge of environmental conditions [24]. A further understanding of how populations in various geographical settings and with different cultural beliefs will increase understanding of other culturally or geographically significant barriers to behaviour change in Canada. This point further emphasizes the importance of framing climate change public health messages with audience and context-specific information to encourage behaviour change and public engagement with the issue.

Even within the greater Hamilton area of the Golden Horseshoe Region, further understanding of how urban, suburban and rural residents perceive climate change would be useful to establish sustainable policy recommendations and public health messages suited to specific populations. The participants of this particular research were primarily urban or suburban community residents, and have thus experienced a largely constructed physical environment that will play a significant role in shaping their experiences of environmental change. While a constructed physical environment carries its own health risks under changing environmental conditions (poor air quality, flooding, extreme heat), perceptions and experiences discussed by participants in this region will be vastly different than residents of other Canadian populations.

## Conclusions

This research has attempted to provide a more in-depth understanding of the knowledge, attitudes and practices associated with climate change and health among residents (n = 22) of the greater Hamilton area of Southern Ontario. The results support recent calls for reframing climate change from an environmental to a public health issue in order to increase public engagement in adaptive and mitigative behaviour change, and to increase support for climate change policy [5,6]. While previous research touched on public awareness of the human health risks of climate change, we have further explored the attitude-action link through the examination of facilitators and barriers to behaviour change.

Understanding how the public understands and behaves in relation to climate change and health risks will be important for policy makers and the public health community in order to educate the public about the serious health issues associated with climate change and to address public-identified barriers to behaviour change. To face the adaptation and mitigation challenges of climate change on a Canadian and global scale, it will be increasingly important for policy makers to recognize and react to public opinion related to global climate change and health as well as expressed need for supported action.

## Competing interests

The authors declare that they have no competing interests.

## Authors’ contributions

FSC and SJE developed the research question. FSC conducted data collection, data analysis and prepared figures and tables. SJE provided feedback and guidance. Both authors contributed to the final draft of the paper. Both authors read and approved the final manuscript.

## Pre-publication history

The pre-publication history for this paper can be accessed here:

http://www.biomedcentral.com/1471-2458/13/208/prepub

## Supplementary Material

Additional file 1 **Study area demographics [**[25]**].** See Additional File 1 for an overview of the sociodemographic variables (such as gender, age, education, and median income) of the study area population. Click here for file

Additional file 2 **Participant demographic breakdown.** See Additional File 2 for a breakdown of the sociodemographic variables (such as the year of birth, gender, marital status, employment status and education) of the research participants (n = 22). Click here for file

Additional file 3 **Interview schedule.** See Additional File 3 for the interview schedule (including questions and specific probes) used in the research. Click here for file
