# Supplementary material for: Making the links: do we connect climate change with health? A qualitative case study from Canada
Source: BMC Public Health. 2013 Mar 8;13:208. doi: 10.1186/1471-2458-13-208 (PMC3608965; doi:10.1186/1471-2458-13-208)
Supplement: Additional file 1 — Study area demographics [[25]]. See Additional File 1 for an overview of the sociodemographic variables (such as gender, age, education, and median income) of the study area population. [file 1471-2458-13-208-S1.docx]

**Additional File 1: Study Area Demographics (Statistics Canada, 2006)**

|  |  | **BURLINGTON** | **HAMILTON (incl. Dundas & Stoney Creek)** | **BRANTFORD** | **OAKVILLE** | **ONTARIO** |
| --- | --- | --- | --- | --- | --- | --- |
|  |  |  |  |  |  |  |
| **Population (2006)** |  | 164,415 | 504,560 | 124,610 | 165,615 | 12,160,285 |
| **Males** |  | 78985 (48.0%) | 245690 (48.7%) | 60470 (48.5%) | 80305 (48.5%) | 5930700 (48.8%) |
| **Females** |  | 85425 (52.0%) | 258870 (51.3%) | 64135 (51.5%) | 85305 (51.5%) | 6229580 (51.2%) |
| **Land Area (km^2^)** |  | 185.74 | 1117.21 | 1072.90 | 138.56 | 907573.82 |
| **Median Age** |  | 40.30 | 39.60 | 39.60 | 38.40 | 39.00 |
| **Immigrants** |  | 36280 (22.3%) | 126485 (25.4%) | 15935 (13.0%) | 50250 (30.5%) | 3398725 (28.3%) |
| **Canadian Citizens** |  | 154270 (94.9%) | 467330 (94.0%) | 119595 (97.4%) | 152210 (92.5%) | 11131465 (92.5%) |
| **No certificate, diploma or degree** |  | 20930 (15.8%) | 102180 (25.1%) | 28365 (28.5%) | 18515 (14.2%) | 2183625 (22.2%) |
| **Unemployment rate (%)** |  | 4.6 | 6.5 | 6.0 | 5.3 | 6.4 |
| **Total visible minority population** |  | 15690 (9.7%) | 67845 (13.6%) | 6715 (5.5%) | 30315 (18.4%) | 2745200 (22.8%) |
| **Median income (15 years and over [$])** |  | 34,379 | 26,353 | 26,703 | 35,650 | 27,258 |
| **Mother Tongue English** |  | 133,020 (81.9%) | 363,115 (73%) | 107,725 (87.7%) | 119,460 (73%) | 8,230,705 (68.4%) |
