# Supplementary material for: Making the links: do we connect climate change with health? A qualitative case study from Canada
Source: BMC Public Health. 2013 Mar 8;13:208. doi: 10.1186/1471-2458-13-208 (PMC3608965; doi:10.1186/1471-2458-13-208)
Supplement: Additional file 2 — Participant demographic breakdown. See Additional File 2 for a breakdown of the sociodemographic variables (such as the year of birth, gender, marital status, employment status and education) of the research participants (n = 22). [file 1471-2458-13-208-S2.docx]

**Additional File 2: Participant Demographic Breakdown**

| **Participant Number** | **Year of Birth** | **Gender** | **Marital Status** | **Children** | **How many?** | **Country of Birth** | **Municipality** | **Employment Status** | **Education** | **Political Views** | **Community Involvement** | **Recruitment** |
| --- | --- | --- | --- | --- | --- | --- | --- | --- | --- | --- | --- | --- |
| 1 | 1983 | F | Single | N | 0 | Canada | Hamilton | Unemployed | Masters | Liberal | Middle | Kijiji |
| 2 | 1968 | F | Divorced | N | 0 | Canada | Dundas | Part-time | College | Middle | Middle | Kijiji |
| 3 | 1968 | M | Single | N | 0 | Canada | Dundas | Full-time | High School | N/A | Not Involved | Kijiji/Friend |
| 4 | 1989 | F | Single | N | 0 | Canada | Hamilton | Student | Some college/ university | Liberal | Not Involved | Kijiji |
| 5 | 1968 | F | Married | N | 0 | Northern Ireland | Hamilton | Full-time/part-time | Masters | Middle | Not Involved | Kijiji |
| 6 | 1980 | F | Married | Y | 1 | Canada | Hamilton | Maternity Leave | University | Conservative | Middle | Kijiji |
| 7 | 1958 | F | Married | Y | 6 | Egypt | Stoney Creek | Full-time | Masters | Liberal | Very Involved | Kijiji |
| 8 | 1955 | F | Married | Y | 2 | Canada | Hamilton | Full-time | University | Middle | Not Involved | Kijiji |
| 9 | 1977 | F | Single | N | 0 | Canada | Brantford | Full-time | University | Middle | Not Involved | Kijiji |
| 10 | 1988 | M | Single | N | 0 | Canada | Brantford | Student/Part-time | University | Middle to Liberal | Middle | Aunt |
| 11 | 1972 | F | Single | N | 0 | Canada | Hamilton | Full-time | University | Liberal | Very Involved | Online |
| 12 | 1988 | M | Single | N | 0 | Canada | Hamilton | Full-time | University | Middle | Very Involved | Kijiji |
| 13 | 1942 | F | Single | Y | 3 | Canada | Burlington | Retired | 2 yrs college | Middle | Not Involved | Poster |
| 14 | 1960s | F | Married | Y | 6 | Canada | Oakville | Part-time | Post-grad | Conservative | Not Involved | Kijiji |
| 15 | 1979 | F | Single | N | 0 | Canada | Stoney Creek | Unemployed | Degree (unspecified) | Conservative | Very Involved | Kijiji |
| 16 | 1943 | F | Married | Y | 3 | Canada | Hamilton | Retired | College | Conservative | Middle | Unsure |
| 17 | 1961 | F | Married | Y | 2 | Canada | Hamilton | Full-time | University | Middle to Liberal | Middle to not involved | Co-worker |
| 18 | 1956 | M | Married | Y | 2 | Germany | Hamilton | Full-time | Some University | Middle | Not Involved | Wife |
| 19 | 1988 | M | Single | N | 0 | Canada | Hamilton | Unemployed | High School | Middle | Very Involved | Friend |
| 20 | 1949 | F | Married | Y | 2 | Germany | Oakville | Full-time | Second year University | Conservative | Middle | Craigslist |
| 21 | 1959 | F | Separated | Y | 1 | Canada | Hamilton | Retired | High School | Liberal | Middle | Snowball |
| 22 | 1950 | M | Married | N | 0 | Canada | Burlington | Retired | Some College | Middle | Middle | Kijiji |
