# Supplementary material for: Making the links: do we connect climate change with health? A qualitative case study from Canada
Source: BMC Public Health. 2013 Mar 8;13:208. doi: 10.1186/1471-2458-13-208 (PMC3608965; doi:10.1186/1471-2458-13-208)
Supplement: Additional file 3 — Interview schedule. See Additional File 3 for the interview schedule (including questions and specific probes) used in the research. [file 1471-2458-13-208-S3.docx]

**Additional File 3: Interview Schedule**

| **Theme** | **Questions** | **Probes** |
| --- | --- | --- |
| Health | So to start off, I’d like us to talk a little bit about the health of Canadians. |  |
|  |  |  |
| Meaning | Could you tell me a little bit about what you think makes a person healthy? | (Possibilities include social, mental, physical, environmental aspects) |
|  |  |  |
|  | And what about what makes a community healthy? | Explain |
|  |  |  |
|  | (Depending on their previous answer, they may have already answered this question). |  |
|  | Do you think health and the environment are linked in any way? | What role do you think it plays in making a person or a community healthy or unhealthy? |
|  |  |  |
|  | So how would you describe your own health compared with other people your own age? |  |
|  |  |  |
|  | What are some of the things you do to keep yourself healthy? | Are there any other reasons you do these things? (Possibilities include being cheap, social benefits, saves time, good for environment [ie. biking/walking instead of driving]) |
|  |  |  |
| Priorities | So going back to your community, what are some health concerns you think are important? | Why is that? |
|  |  | Don’t worry if you have already mentioned some of these things, you can repeat yourself. |
|  | Is there anything you would change in your community to make it a healthier place for you/your family? |  |
|  |  |  |
| Environment | Now I’d like us to talk a little bit about the environment. |  |
|  |  |  |
|  | What do the words global environmental change mean to you? | Explain |
|  |  |  |
|  | What about climate change? | Do you think there’s a difference between climate change and global warming? |
|  |  |  |
|  | Do you think the average person understands? |  |
|  |  |  |
|  | Did you ever study the environment in school? | What in particular? |
|  |  |  |
|  | Other than in school have you heard these terms mentioned anywhere else? | Explain |
|  |  |  |
|  | What sorts of things can people do to help the environment? | Possibilities include: Recycle/Green Bin, Car pool, Public transit, Biking, Unplugging/Switching off lights, Water bottles, Purchase local/in-season produce, decrease home energy use, Reducing use of spray cans |
|  |  |  |
|  | What do you do? |  |
|  |  |  |
|  | (If they explain how they behave in environmentally friendly ways…) Why do you think people should do these things? | Possibilities include: out of concern? Altruism? Feel obliged/that it's the 'right' thing to do? |
|  |  |  |
|  | And do a lot of people you know/in your community _____ (act in these ways)? | Explain |
|  |  |  |
|  | Do you think anything would encourage or discourage people from acting in more ‘environmentally friendly’ ways? | Possibilities include: Save time, save money, convenience, more time outdoors, why/why not? |
|  |  |  |
|  | Could you tell me about any environmental organizations or community programs or resources related to environmental issues that you know of offered in your community? | (For example, Royal Botanical Gardens, Environment Hamilton, Bruce Trail Association, Conservation areas, library resources, etc…) |
|  |  |  |
|  | Are you involved in any of these programs? | Why/why not? |
|  |  |  |
|  | Are you satisfied with what you do to help the environment? | Why/why not? Would you change anything? If so, what? |
|  |  |  |
|  | Could you explain to me any reasons these changes couldn’t be made? | Possible barriers could include: inability to change (money), lack of knowledge, fatalist attitude, skepticism/uncertainty, do not feel accountable, distrust (of information sources), saturation (I'm already doing everything I can do), inconvenience (time), self-interest, lack of motivation |
|  |  | Is there anything else? |
|  |  |  |
| Conclusion | Thank you very much for taking the time to participate in my research today. It has helped me have a better understanding of how you perceive health and the environment. |  |
|  |  |  |
|  | You have expressed that you feel (brief summary), is there anything else you would like to add? |  |
